# A Case Report on the Rare Presentation of the Primary Spinal Epidural Non-Hodgkin's Lymphoma

**DOI:** 10.7759/cureus.28833

**Published:** 2022-09-06

**Authors:** Shobha Mandal, Mary Grace Bethala, Dipesh K Rohita, Sarah A Branch, Phillip Lowry

**Affiliations:** 1 Internal Medicine, Guthrie Robert Packer Hospital, Sayre, USA; 2 Internal Medicine, GlobeHealer, Philadelphia, USA; 3 Internal Medicine, B.P. Koirala Institute of Health Sciences, Dharan, NPL; 4 Medicine, Geisinger Commonwealth School of Medicine, Scranton, USA; 5 Hematology/Oncology, Guthrie Robert Packer Hospital, Sayre, USA

**Keywords:** primary spinal epidural non-hodgkin's lymphoma, cns tumor, radiotherapy, chronic back pain, diffuse large b-cell lymphoma

## Abstract

Primary spinal epidural non-Hodgkin's lymphoma (PSENHL) is a tumor of central nervous system origin. It is one of the rarest tumors seen in the fourth to fifth decades of life. The majority of PSENHLs are diffuse large B-cell lymphomas and are most commonly caused because of chronic inflammatory process, chronic infection, or autoimmune disease. Here, we are presenting a case of a 51-year-old male who was found to have a diffuse large B-cell lymphoma, specifically germinal center B-cell type that is considered a rare presentation.

## Introduction

Primary spinal epidural non-Hodgkin's lymphoma (PSENHL) is one of the rare central nervous system (CNS) tumors. Among all the central nervous system tumors, only 0.1%-6.5% arise from the epidural location. The majority of PSENHLs are diffuse large B-cell lymphomas and are usually seen in individuals 40-50 years of age. PSENHL affects the midthoracic spine (69%), lumbar spine (27%), and cervical spine (4%) [1,2].

## Case presentation

A 51-year-old gentleman presented with a complaint of worsening back pain radiating to the right lower quadrant of the abdomen for the last four months. His past medical history was significant for tobacco use, hypertension, and a traumatic back injury 16 years prior that was treated with intra-articular steroid injections. The pain was dull, 7/10 in intensity, worsened with movement, and was minimally relieved with analgesics. Despite the injections, the back pain continued.

**Figure 1:**
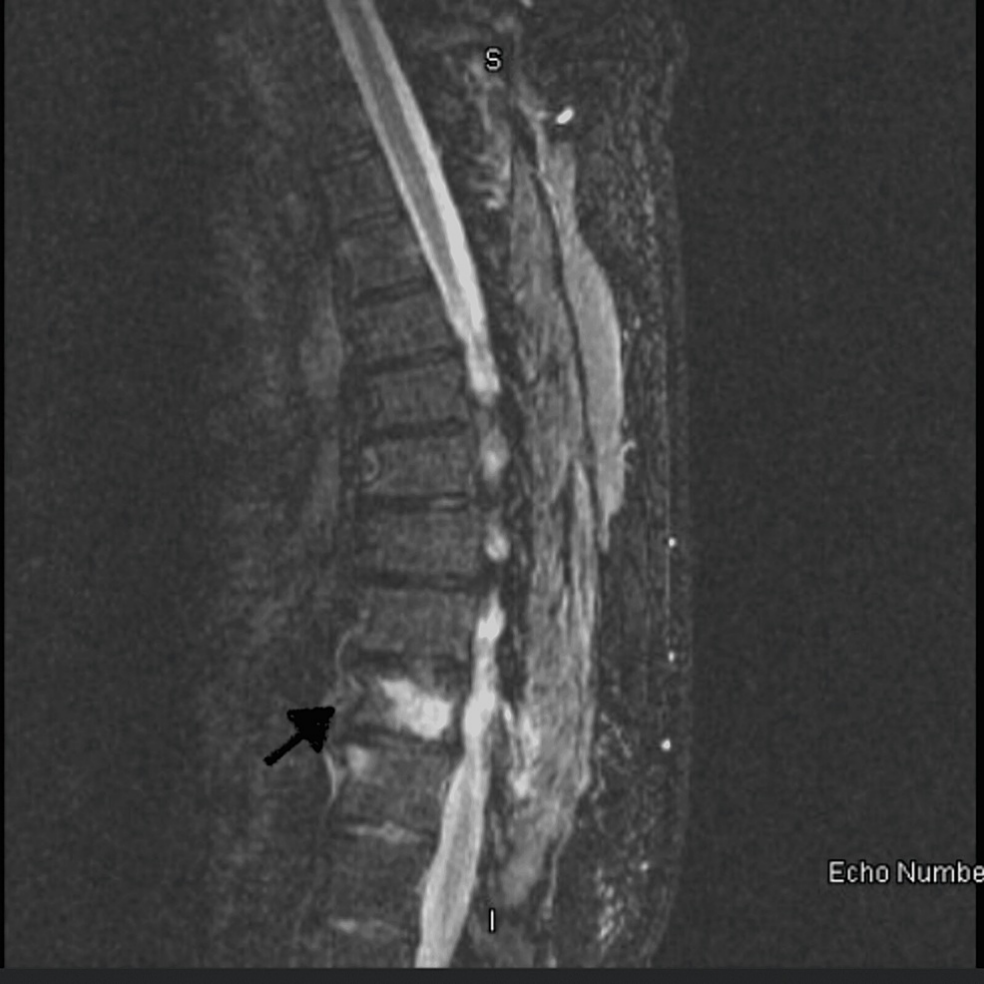
MRI of the spine with T10 vertebral body enhancement with abnormal signal focus, labelled with the black arrow (sagittal view)

**Figure 2:**
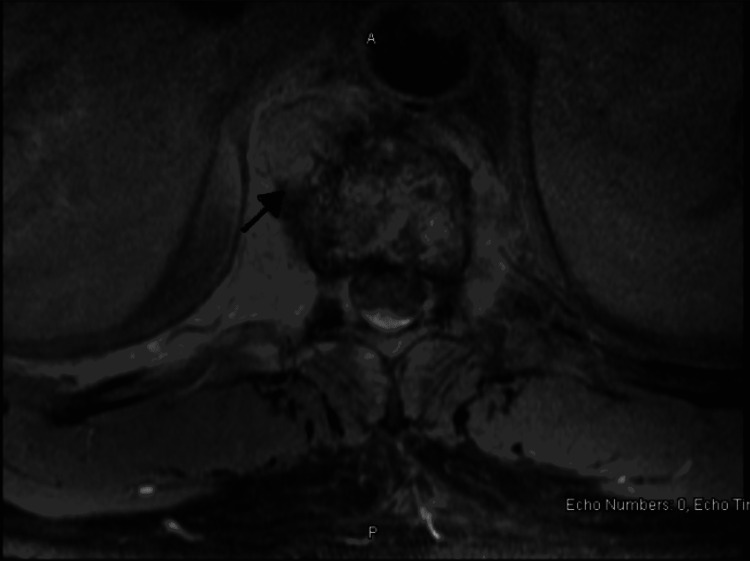
MRI of the spine with T10 vertebral body enhancement with abnormal signal focus, labelled with the black arrow (axial view)

On examination, the patient was vitally stable, and the physical exam was within normal limits except for mild tenderness to palpation in the right lower quadrant of the abdomen. The neurologic examination and anal sphincter tone were normal. The laboratory work-up was significant for leukocytosis, but an X-ray of the spine was within normal limits. Magnetic resonance imaging (MRI) of the spine revealed enhancement of the T10 vertebral body with a 0.5 cm (about 0.2 in) abnormal signal focus suggestive of an abscess, spondylitis, or neoplastic process (Figures 1, 2).

**Figure 3:**
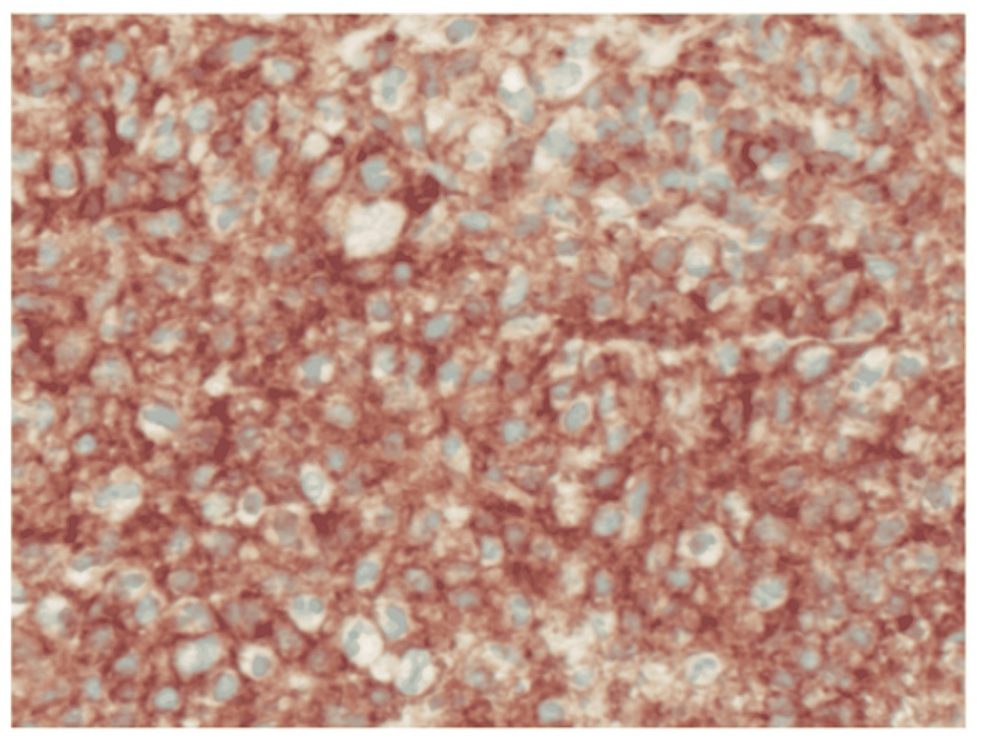
Sheets of large atypical lymphocytes, magnification x400

**Figure 4:**
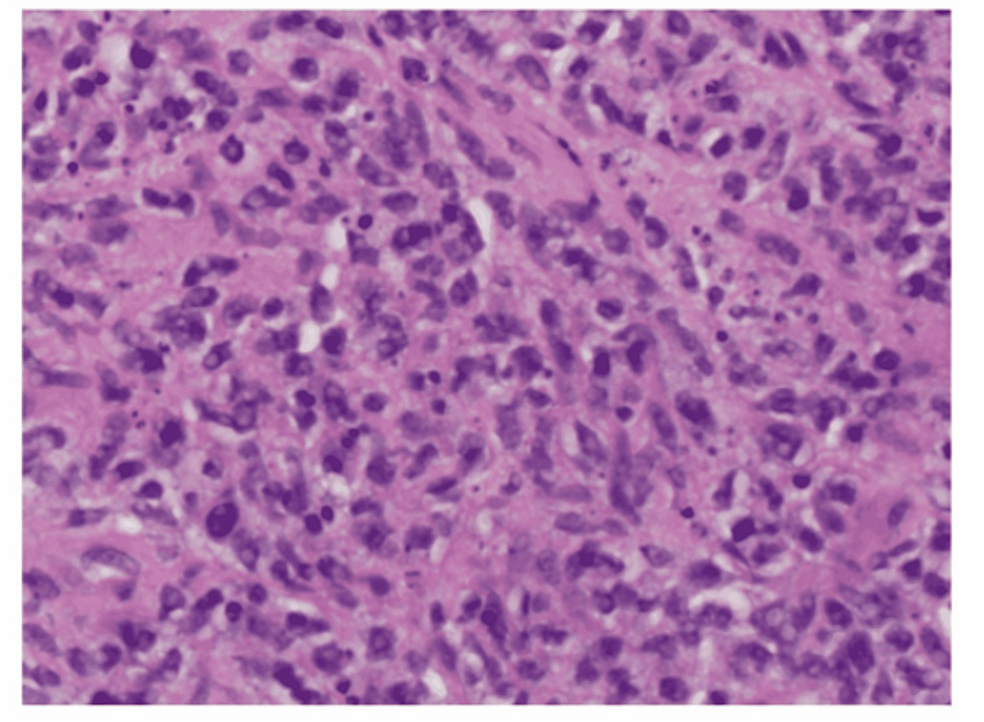
Immunohistochemistry showing lymphocytes positive for CD10, magnification x400

Believing the cause might be infectious, the patient was initially started on empiric antibiotics of IV ceftriaxone 2 g daily and vancomycin. The blood culture did not show any growth, and so, the antibiotics were subsequently stopped. The patient was then suspected of having a tumor and underwent a computed tomography-guided aspiration biopsy of the mass that was negative for infection and malignant cells. The patient was evaluated by a neuro-oncology surgeon and underwent a T11 laminectomy with total resection of the mass. The mass was rubbery, firm, grayish in color and adherent to epidural space. The final histopathological examination showed diffuse a large B-cell lymphoma, specifically germinal center B-cell type (Figure 3). Immunohistochemistry was LCA+, CD10+, CD20+, CD 79a+, BCL 6+ with a Ki-67 proliferation index positive in 80% of neoplastic cells (Figure 4), and negative for CD3, CD5, CD43, BCL-2, MUM1 and cyclin D1. Immunostaining was negative for CD34, CD 68, S100, pan keratin, vimentin, and Mart 1. Double-hit lymphoma studies were negative. FISH was negative for the rearrangement of MYC or BCL6 and was negative for IGH-BCL2 fusion.

The final diagnosis of the primary spinal epidural diffuse large B-cell lymphoma was made. Postoperatively, his back pain improved and he was started on the prednisone taper of 80 mg x 4 days, 40 mg x 1 day, 20 mg x 2 days, and then 10 mg x 1 day. The oncology department planned treatment with systemic chemotherapy followed by consolidation radiation therapy to the lower thoracic spine. If the positron emission tomography (PET) scan shows no evidence of disease anywhere other than the spine, then three cycles of chemotherapy followed by radiation would be done. However, if there is evidence of disease elsewhere, then more cycles of systemic chemotherapy would be indicated. Since there was spine involvement, it was recommended to follow with consolidation radiation therapy after the completion of chemotherapy. The PET scan showed the fluorodeoxyglucose (FDG) uptake in the T10 and T11 vertebral bodies, adjacent right paraspinal and intercostal soft tissues, as well as an increased uptake in mediastinal lymph nodes, concerning malignancy. Bone marrow was also performed, and the aspirate and biopsy showed no evidence of tumor involvement. The patient also underwent a lumbar puncture with cerebrospinal fluid (CSF) analysis, and cytology was negative for malignancy. He was then started on chemotherapy, and is now status negative following four cycles of R-CHOP (rituximab-cyclophosphamide-hydroxydaunorubicin-Oncovin-prednisone regimen) and two cycles of intrathecal methotrexate. The repeat MRI of the thoracic spine with and without contrast did not show any recurrent epidural mass, and there was even an interval decrease in the paraspinal tumor size. He also denied any complaint of tiredness, weight loss, shortness of breath, bowel and bladder dysfunction, or weakness in his lower extremities. In this case, the patient had significant improvement in his back pain after decompressive surgery and postoperative radiation therapy. He is currently being treated with chemotherapy and is tolerating it well. He does not have any neurological deficits or symptoms of bowel/bladder dysfunction.

## Discussion

Primary spinal epidural non-Hodgkin’s lymphoma is one of the rarest CNS tumors. The diffuse large B-cell lymphoma involving the spinal epidural space could be further classified into germinal center B-cell type, which is CD10+ or CD10-, BCL-6+, MUM1- and non-germinal center B-cell type that can be CD10-/BCL-6- or CD10-/BCL-6+/MUM1- type [3]. It most commonly affects the midthoracic spine (69%), the lumbar spine (27%), and the cervical spine (4%) [1,2].

Lymphomas are malignant lymphoid tumors that usually develop in the lymphoid tissues and can spread to other organs. Less commonly they can arise from extra-nodal locations like the skin, tongue, lung, stomach, small bowel, and thyroid [4]. Extra-nodal non-Hodgkin's lymphomas (NHLs) account for about 24%-48% of all NHLs [5]. Extra-nodal NHLs involving only the epidural space account for 3.3% of all lymphomas, 9% of all the epidural spinal tumors, and 0.9% of all extra-nodal NHLs [6].

The exact etiology of PSENHLs is unclear, but it is assumed that autoimmune diseases, chronic inflammatory processes, and chronic infections may play a role [5]. The lymphoma may present with chronic back pain or radicular pain further progressing to spinal cord compression that can result in neurologic deficits such as paresis, ataxia, and/or sensory disturbance with bowel and bladder incontinence. Patients may present with two phases of symptoms. In the first prodromal phase, presentation can be local pain in the back accompanied by radicular pain in the legs and abdomen persisting for several months to one year. This is followed by the second phase, marked by spinal cord compression within two to eight weeks [7]. As most of the patients present with the complaint of chronic back pain, it most frequently is misdiagnosed as pathological compression fractures, infection or abscess of spine, or metastatic disease; misdiagnosis delays appropriate management. In this case also, the patient was in the first phase of clinical presentation, which could have progressed to cord compression, as he was having on-and-off back pain for more than six months.

All patients presenting with the complaint of chronic back pain should be evaluated thoroughly as it is difficult to diagnose underlying pathologies based on a clinical examination alone. The exclusion of a primary lymphoma requires a proper laboratory work-up for inflammatory markers, radiological imaging with myelography, computed tomography, as well as MRI [8]. Laboratory work-up including routine investigations, such as erythrocyte sedimentation rate peripheral blood smear and blood culture, should also be included. Furthermore, patients should be investigated with a bone marrow biopsy and CSF analysis and cytology for diagnosis, as CSF abnormalities have been found in 97% of patients of NHL with CNS involvement [9]. The sternum and iliac crest are the most common sites used for bone marrow aspiration for biopsy to rule out lymphoreticular involvement.

Due to the rarity of the tumor, guidelines for the management of PSENHLs are not certain. Emergency decompressive surgery, with or without resection, in the acute phase followed by radiotherapy and chemotherapy is considered as the mainstay of treatment of PSENHLs. Any patient with acute paresis and/or loss of bladder/bowel control requires emergency decompression of the spinal cord [5]. The surgical approach can vary depending on the location of the tumor, the degree of spinal cord compression, spinal instability, and the patient's general condition [5]. Spinal irradiation and systemic chemotherapy are important adjuvant treatments, which have been shown to increase disease-free survival. Intrathecal chemotherapy can also be considered in cases of relapse [10]. Post-surgery radiotherapy combined with chemotherapy agents of cyclophosphamide, vincristine, and prednisone have been recommended for patients with spinal involvement [11].

Earlier diagnosis and treatment are associated with improved functional outcomes [11]. It has been found that the prognosis for functional recovery in patients with spinal cord compression due to an epidural NHL is better than that of patients with metastatic carcinoma [10]. When treated early with surgery and multidisciplinary treatment, patients are found to be curable [11].

## Conclusions

PSENHLs can be missed in patients who present with symptoms such as worsening back pain. Given the fact that PSENHLs are so rare, the diagnosis becomes more challenging. In any patient who has progressively worsening back pain with any neurological signs and symptoms, a high index of clinical suspicion is needed to consider this rare condition. Timely diagnosis and treatment are vital to improve the functionality, outcomes, and quality of life.
